# Probiotics for human health –new innovations and emerging trends

**DOI:** 10.1186/1757-4749-4-15

**Published:** 2012-11-26

**Authors:** Sunita Grover, Hogarehalli Mallapa Rashmi, Anil Kumar Srivastava, Virender Kumar Batish

**Affiliations:** 1 Molecular Biology Unit, Dairy Microbiology Division, National Dairy Research Institute, Karnal, Haryana, 132001, India

**Keywords:** Gut microbiota, Probiotics, Clinical evidence, Health benefits

## Abstract

The role of the gut microbiome in human health and disease with a particular emphasis on therapeutic use of probiotics under specific medical conditions was mainly highlighted in 1st Annual conference of Probiotic Association of India (PAi) and International Symposium on “Probiotics for Human Health - New Innovations and Emerging Trends” held on 27th-28th August, 2012 at New Delhi, India. There is increasing recognition of the fact that dysbiosis or alteration of this gut microbiome may be implicated in gastro-intestinal disorders including diarrheal diseases, ulcerative colitis, inflammatory bowel diseases, life style diseases viz. Diabetes Mellitus-2 and obesity etc. This report summarizes the proceedings of the conference and the symposium comprehensively. Although, research on probiotics has been continuing for the past few decades, the subject has been currently the major focus of attention across the world due to recent advances and new developments in genomics, transcriptomics, proteomics, metabolomics and emergence of new generation of high through put sequencing technologies that have immensely helped in understanding the probiotic functionality and mode of action from nutritional and health perspectives. There is now sufficient evidence backed up with good quality scientific clinical data to suggest that probiotic interventions could indeed be effective in various types of diarrheal diseases, other chronic gastrointestinal inflammatory disorders like pouchitis, necrotizing entero-colitis, allergic responses and lactose intolerance etc. This report makes a modest attempt to give all the stake holders involved in development of probiotic based functional/health foods an overview of the current status of probiotics research at the Global and National level. The most crucial issues that emerged from the lead talks delivered by the eminent speakers from India and abroad were the major focus of discussions in different plenary and technical sessions. By discussing some of these issues from scientific perspectives, the conference could achieve its prime objective of disseminating the current knowledge on the prospects of probiotics as potential biotherapeutics in the management of human health and diseases.

## Introduction

Probiotics constitute the new buzz word in human dietary portfolio and are currently the major focus of attention across the world including India and other developing counties due to their enormous health potentials. Our understanding of the functionality of human gut microbiota and increasing use of probiotic organisms specifically lactobacilli and bifidobacteria as functional microbial dietary ingredients for promoting human health have made considerable impact on the consumers. As s result of recent advancements in the science of probiotics in terms of efficacy, mode of action, probiotic- gut microbiota-host interactions at molecular level with the help of high through put sequencing techniques and nutrigenomics, the application of probiotics offers an innovative approach for development of novel probiotic formulations under the category of functional foods for the management of specific diseases particularly chronic inflammatory gastrointestinal disorders and other medical conditions. However, specific health claims associated with probiotics/ probiotic food formulations and their safety through scientific evidence based clinical studies in the target human population remain a real challenge to establish the credibility of their health promoting functions. Nonetheless, this limitation does not undermine the immense power of probiotics to promote human health and confer protection against diseases besides introducing lot of value to the foods from nutritional perspectives. In view of the significant impact, these invaluable food-grade microbial-ingredients are likely to make on nutritional well being and public health, it was considered highly appropriate to do some brain storming on the most critical emerging issues. Some of the pertinent issues related to probiotic functionality which need immediate attention for in depth discussions at an open platform include efficacy and safety of probiotics, establishing their health claims against specific diseases in the target subjects both *in vitro* and *in vivo* models, their mode of action and validation in well designed clinical studies, probiotic product development, effective dosage, stability and survival of probiotics during processing and storage and regulatory issues etc. Addressing these issues holistically through mutual discussions with all the concerned stake holders could pave way in developing a road map for future endeavors on these lines in the effective usage of probiotics for the welfare of the society in the country. Realizing the enormous prospects and significance of probiotics in human health in the backdrop of the aforesaid issues from global perspective and the tremendous feedback received from the industry, the concept of Probiotic Association of India (PAi) was mooted and subsequently the same was got registered as a society with effect from Dec. 8, 2010. Backed up with more than 200 registered members represented by faculty and students from academic organizations and industry as corporate members, PAi initiated the process of organizing its first annual conference from a broader perspective to give a global touch to this scientific event by clubbing it with an International symposium with the theme title “Probiotics for Human Health- New Innovations and Emerging Trends”. Accordingly, the 1st Annual Conference of PAi and International Symposium on “Probiotics for Human Health- New Innovations and Emerging Trends” was organized on 27th-28th August, 2012 at India Habitat Centre, Lodi Road, New Delhi. The conference was attended by about 200 participants from different parts of India and abroad. The eminent speakers invited for delivering their lead papers in their respective areas of interest in probiotic research in different plenary and scientific sessions of the conference included seven from abroad representing Finland, Spain, Ireland, UK/Scotland, Australia, Denmark and Hong Kong and nine from India. Three of the overseas speakers in the conference were nominated by International Scientific Association of Probiotics and Prebiotics (ISAPP). The proceedings of the two day conference as per the program schedule are summarized below.

### Session on oral presentations by young researchers

The conference began with a session on oral presentations by young researchers to present and exchange new data and cutting edge ideas in the field of probiotics. A total of six young researchers presented their recent work on probiotics in their respective areas of research and their performance was monitored by an expert panel of three judges comprising of Dr. C.S. Yajnik, Director, Diabetes Unit King Edward Memorial Hospital, Pune, India; Dr. Karen Scott, Rowett Institute of Nutrition and Health, Scotland, UK and Dr. Nagendra Shah, Professor of Food Science, University of Hong Kong, Hong Kong. Ms Suja Senan from GAU, Anand, Gujarat, the first speaker in the session presented her work on the annotation of complete whole genome sequence of *Lactobacillus helveticus* MTCC 5463 [1], the indigenous probiotic strain (claimed to exhibit significant antimicrobial activity, lowering of cholesterol in humans along with expressing positive immuno-modulating effects) from safety perspective. She particularly targeted antibiotic resistance, production of harmful metabolites, potential virulence factors, biogenic amines, D-lactic acid, azoreductase and nitroreductase as the potential carcinogenic biomarkers at the gene sequence level to dwell on their role in the safety of the aforesaid strain. Based on the complete whole genome sequence analysis, she concluded that a detailed bio-informatic analysis related to biosafety of probiotic strain MTCC 5463 could provide very useful information on its genomic stability, antibiotic resistance, virulence and production of harmful metabololites. While addressing the concerns raised over the possibility of antibiotic resistance transfer to the gut microbiota, the absence of mobile genetic elements and chromosomally-encoded antibiotic resistance mechanisms in this indigenous probiotic strain, Ms Suja stated that *in-silico* analysis of the whole genome sequence could satisfy the queries with regard to its overall safety.

Ms Ruchi Vaidya from M. S. University of Baroda, Vadodara, Gujarat made a presentation of her recent work on the outcome of a cross sectional study designed to determine the associations of gut microbiota on glucose and lipid response in Type 2 diabetic patients. While sharing the data on the key findings of this study, she revealed that almost 77% of the subjects had poor control of diabetes as could be reflected from high values of HbA_1c_ FBS and PP_2_BS concentrations in the blood. The mean cholesterol and triglyceride levels of the subjects were also on the higher side. Almost 62% of the subjects had considerably high LDL and 61% had poor HDL levels. While commenting upon the changes in the gut microflora in the subjects of the study, she further stated that there was heavy colonization of Lactic acid bacteria and *Bifidobacteria* that showed a significant negative association with HbA_1c_, TG, VLDL and TC/HDL ratio (p<0.05) along with BMI. Fat intake of the subjects also showed a strong negative association with fecal LAB and bifidobacterial counts and a positive association with enteric pathogens where as fibre intake and intake of probiotics and prebiotic rich foods showed a vice versa associations with the fecal microbial counts. Lactic acid bacteria and bifidobacteria were significantly low in subjects with presence of 3 or more diabetic complications where as enteric pathogen counts were higher. She concluded that the type of microbiota established in the gut plays a significant role in arresting the complications of Type 2 DM.

Dr Raj Kumar Duary from Tezpur University, Napaam, Assam spoke about his work on the functional efficacy of two indigenous probiotic *Lactobacillus plantarum* strains with regard to their acid and bile tolerance, hydrophobicity, adherence property, role of surface layer proteins in colonization, mucosal barrier function, anti-inflammatory and immuno-modulatory effects on HT-29 cells. He briefed the audience that after mass screening of indigenous putative probiotic lactobacilli based on their hydrophobicity and cell adhesion property on HT-29 and Caco-2 cell lines, Lp9 and Lp91 were shortlisted for quantification of relative expression of the genes involved in acid and bile tolerance along with colonization potentials under *in vitro* conditions simulating gut environment as well as their immuno-modulatory functions in HT-29 cells by RT-qPCR [2]. Lp91 exhibited relatively higher acid tolerance by up-regulating the relative expression ‘*atp*D*’* gene at pH 2.5 after 90 min [3]. Expression of ‘*bsh’* gene was also up-regulated maximally in both the selected strains under 2% bile concentration with maximum attained with Lp91 [4]. In the context of surface proteins, ‘*mub’* gene was maximally expressed in Lp9. Almost, the same trend was recorded in the expression of ‘*map*A*’* in Lp9. ‘EF-Tu’ gene, on the other hand, was expressed highest in Lp91. However, there were inter strain variations in respect of the expression of all the three surface proteins. Both the probiotic strains demonstrated strong immuno-modulatory and anti-inflammatory properties by up regulating MUC2, anti-inflammatory cytokines and other signaling molecules [5]. Lp91 was rated as the most effective by significantly up regulating IL-10 and IFN-α expression in HT-29 cells under pre-culturing conditions. He finally concluded that Lp91 and Lp9 were the most promising indigenous probiotic strains and have the prospects to be explored as potential biotherapeutics against inflammatory disorders.

Mr. Umesh Kumar from NDRI, Karnal was the next young researcher who presented his work on the anti-allergic effect of dahi made with *Lactobacillus acidophilus* and *Bifidobacterium bifidum* in mice. While presenting his data, he informed that feeding of probiotic dahi significantly suppressed (p<0.001) the elevation of whey proteins-specific IgE and IgG response in the serum of WP-sensitized mice. In addition, sIgA levels were significantly (p<0.001) increased in intestinal fluid collected from mice fed with La-Dahi or LaBb-Dahi. Production of Th-1 cell-specific cytokines i.e. interferon-γ (IFN-γ), interleukin-12 (IL-12) and IL-10 increased; while Th2-specific cytokines (IL-4) decreased in the supernatant of 48 hr cultured splenocytes collected from mice fed with probiotic dahi compared to the other groups. Moreover, the splenic mRNA levels of IFN-γ, interleukin-10 were significantly increased with concomitant decrease in the expression of IL-4 in La-Dahi or LaBb-Dahi groups, as compared to control groups. From the outcome of this study, he concluded that probiotic dahi suppressed WP-induced allergic consequences characterized by decreasing levels of IgE, IgG in serum and increasing intestinal sIgA levels. Cytokines profile also indicated the shift from skewed Th2-pathway to cytotoxic Th1-pathway for attaining balance.

Mr. Himanshu Kumar from NCCS, Pune in his presentation focused on exploring the microbial diversity of Kutajarista, an Indian Ayurvedic preparation in quest for finding novel probiotic strains. He demonstrated that microbial diversity in Kutajarista increased after 8th day of fermentation but gradually decreased with only 3 operational taxonomic units (OTU’s) recovered at the saturation of fermentation. Amongst the microbial types, *Lactobacillus* spp. was found at initial time point of fermentation. When assessed for probiotic attributes, *Lactobacillus plantarum* isolated from Kutajarista was highly resistant to acidic pH 2, 0.3% bile concentration and simulated gastric juice [6]. *L. plantarum* also adhered HT-29 cells and colonized mouse gut as revealed by FACS and confocal analysis. He further stated that the maximum colonization of the probiotic *Lactobacillus plantarum* occurred at colonic part of the intestine. The antibacterial activity of *L. plantarum* against enteric pathogens was also demonstrated along with amelioration of cytotoxicity caused by *Aeromonas veronii* in Vero cell line. Besides this, disruption of tight junction proteins in MDCK cell line induced with the same pathogen was prevented by *L. plantarum*. Immuno-modulatory and anti-inflammatory roles of *L. plantarum* were also demonstrated in mouse macrophage RAW cells activated by *A. veronii*. *Lactobacillus plantarum* strain from Kutajarista was stable for 14 days in comparison to 8 days with commercial GG strain. Furthermore, the viability of *Lactobacilli* in Kutajarista was found to be enhanced further when co-cultured with *Saccharomyces cerevisiae* isolated from the same product suggesting specific interaction between these strains which helps in increased survival in Kutajarista matrix. This study highlights the potential use of alternative sources like ayurvedic preparations for isolation of indigenous probiotic microbes. Kutajarista has unique therapeutic properties for gastrointestinal disorders and therefore, it could be an excellent vehicle for incorporation of these strains.

Mr. Satvinder Singh from NDRI, Karnal was the last speaker in the session to present his findings on the anti-diabetic potential of two *Lactobacillus rhamnosus* strains against type 2 diabetes in a rat model. Diabetes was induced in rats fed with high fat diet followed by streptozotocin and divided into three groups viz. two probiotics and one control. He presented the data on the antioxidative effects of the two strains in terms of total anti-oxidative property (TBARS) and activities of anti-oxidative enzymes viz., catalase, superoxide dismutase (SOD) and glutathione peroxidase (GPx) in liver and erythrocytes. Besides this, expression of proglucagon and prohormone convertase1 (PC1) genes in cecum, and TNF-α, IL-6 and adiponectin in epididymal fat were also investigated by real-time PCR. Amongst the two experimental groups based on two distinct *Lactobacillus rhamnosus* strains, HFD+17 group had improved oral glucose tolerance test, lower FBG, GHb, free fatty acids, triglycerides, total cholesterol, LDL-cholesterol and atherogenic index, and higher plasma insulin and HDL-cholesterol after 3 and 6 weeks of feeding. Even though the total number of bacteria was same in the two experimental groups, HFD+17 had more bifidobacteria and lactobacilli in comparison to HFD+LGG. Relative propionate proportions (%) were significantly lower in case of HFD+17 than HFD+SM and HFD+LGG groups. HFD+17 group had significantly lower TBARS and increased activity of catalase, SOD and GPx in liver and erythrocytes. Similarly, HFD+17 group showed increased expression of proglucagon and PC1 and decreased expression of TNF-α and IL-6 genes. It can be concluded from the study that *L. rhamnosus* NCDC 17 does have anti-diabetic effect through enhancing the beneficial bacteria, activities of anti-oxidative enzymes and expression of incretin hormone, and lowered propionate, TBARS and expression of inflammatory markers in diabetic rats.

### Inaugural session

The conference inaugural session began with the welcome address given by Dr. A. K. Srivastava Director, National Dairy Research. Institute, Karnal and President, Probiotic Association of India. Dr. Srivastava welcomed the delegates and apprised them with the theme of the International symposium on probiotics. He presented a comprehensive overview of the conference scientific programme with major focus on multi-dimensional role of probiotics in human health and well being. Dr. Srivastava comprehended that probiotics as functional foods, dietary supplements and bio-therapeutics are becoming increasingly popular amongst the health conscious consumers, health care professionals besides the research scientists across the world as the scientific evidence continues to accumulate on their enormous health potentials along with their possible mode of action. The continuing emergence of clinical evidence for benefits to consumers and the subsequent marketing power of these microbial ingredients have now seen probiotics becoming the fastest growing category of functional and health foods. By virtue of expressing a wide spectrum of novel physiological functions beneficial to human health, probiotics are currently being recognized as the grey area of intensive investigations at the global level to derive their beneficial health effects maximally for the welfare of society from nutritional and health perspectives. However, he cautioned that before seeking any specific health claim for any medical condition attributed to a particular probiotic strain or the food formulation developed with the same, it must be validated through well designed double blind placebo controlled clinical studies at least at two differently located centers.

The conference was formally inaugurated by Mr. T. Chandramouli, Chairperson, FSSAI, who in his inaugural address highlighted the initiatives being taken at FSSAI for regulating the quality, efficacy and safety of processed foods including the value added functional foods particularly dairy based enriched with probiotics to confer their beneficial effects on human health and well being. He called upon the delegates and the probiotic fraternity in the country to deliberate at length on the key issues and challenges before the probiotic industry and suggest possible solutions to address these problems for judicious and effective usage of probiotics in the country for the benefit of the consumers and the society through their innovative R&D initiatives. He also sought the help of distinguished scientists having adequate expertise in probiotic research to advise the food authority how to sustain the quality, efficacy and safety of probiotic formulations in food format and as supplements in the Indian market in the backdrop of many spurious probiotic products with false claims entering the Indian market. Dr. Seppo Salminen from Finland also spoke on the immense value of probiotics for health applications. Dr. G. P. Talwar also shared his views on the prospects of exploring probiotic therapy in vaginal health to further extend the scope of probiotic applications. In the end, Dr. V. K. Batish, Secretary, PAi and the Organizing Secretary of the conference proposed a vote of thanks to all the delegates and expressed his deep sense of gratitude to the invited speakers for agreeing to present their key-note addresses and lead papers in the conference.

### Probiotics - from traditional use to scientifically documented health benefits

Probiotics have a long global history of traditional use [7]. The tradition of using probiotic microorganisms for health benefits is now backed by strong scientific evidence with promising strains of probiotics in the prevention and treatment of diseases [8]. Although promising, many probiotics are hindered by inherent physiological and technological weaknesses. Thus, new scientific innovations with meta-biotechnlogy and patho- biotechnology approaches in the screening and designing of probiotics for particular health benefits may provide strong scientific support for future probiotic research. To deliberate on this particular topic, Dr. Seppo Salminen, Professor and Director, Functional Foods Forum, University of Turku, Finland was invited to deliver the key note address covering the entire journey of probiotics starting from basic probiotic concept, definition, brief history of probiotics, their origin, foods as a carrier of probiotics, their safety and efficacy along with regulatory issues and future perspectives. The main focus of his address was on proving specific health claims associated with probiotics through well designed scientifically driven evidence based clinical studies to enhance their credibility in terms of their claimed functional efficacy. He highlighted the importance of microbiota acquisition from mothers prior to or during delivery that provide first stimuli for the maturation of the intestinal immune system and development [9] and conveyed that establishment of microbiome contributes to the maintenance of intestinal homeostasis and mucosal barrier function and any aberrations in that may lead to non-optimal microbiota and predispose them to subsequent diseases. Elaborating his statement, Dr. Salminen stated that early establishment of a healthy gut microbiota hallmarked by that of a healthy breastfed infant, provides the key to long-term well- being. Furthermore, breastfed infants have less allergies, diarrhea, respiratory and gastrointestinal infections due to the characteristics composition and properties of breast milk including presence of high levels of bifidobacteria and lactic acid bacteria [10]. The predominance of bifidobacteria has an important impact on later health and promotion of such microbiota has been taken as a target for nutritional intervention in infants. He also cautioned that although the probiotic science has developed rapidly and the technological and health properties of many probiotics are well defined yet, there is a need to evaluate the impact of manufacturing and food matrices on stability of probiotic properties and quality. Speaking about the limitations of the existing probiotic strains available in the market, Dr. Salminen opined that new probiotics are still required for enhancing the resilience of healthy microbiota and also emphasized that the quest for novel strains should continue to address other targets such as allergy prevention, alleviation of obesity development, dental caries risk reduction, irritable bowel syndrome symptom relief, reduction in the risk of respiratory tract infections and prevention of necrotizing enterocolitis etc. These initiatives also require new human intervention studies for establishing health claims for probiotics and advocating their use as nutritional adjuncts to other therapies [11].

### Gut microbiota in human health and disease

Trillions of microbes inhabit the human intestine, forming a complex ecological community that influences normal physiology and susceptibility to disease through complex host microbial interactions [12]. The distortion in the gut microbial composition may lead to chronic diseases such as autoimmune diseases, colon cancers, gastric ulcers and life style diseases like obesity, type 2 diabetes and cardiovascular disease [13]. To ponder upon this crucial issue, Dr. F. Guarner, Consultant of Gastroenterology, Digestive System Research Unit, University Hospital Vall d’ Hebron, Barcelona, Spin, while delivering his key note presentation spoke on “Gut Microbiota and Digestive Health”. Deliberating the importance of probiotics in human health, he discussed the metabolic, defensive and trophic functions of gut microbiota to host health. He emphasized that it is the host that provides habitat and nutrition to microbial communities: in fact, we feed gut microbiota. Microbial symbionts have been evolutionary adapted to provide the required organic compounds (essential amino acids, vitamins) and the ability to obtain energy from different sources. Further highlighting the importance of commensal gut microbiota in human health and its impact on shaping the immune defense mechanisms in the host, Dr. Guarner threw some light on how gut microbes play an essential role in the development of a healthy immune system as could be evidenced from studies in the germ-free animals. Reiterating his view point on this issue based on the findings of some recent studies, he enlightened the audience that some gut commensals play a major role in the induction of regulatory T cells in the gut lymphoid follicles [14]. The outcome of these studies clearly suggests that control pathways mediated by regulatory T cells are essential homeostatic mechanism by which the host can tolerate the massive burden of innocuous antigens within the gut or on the body surfaces without resulting in inflammation [15]. Continuing further, Dr. Guarner contended that besides several other factors, foods and dietary fibers have a significant impact on the gut microbial ecosystem in terms of both composition and functions and stated that several facts support this notion. Non-digestible carbohydrates and proteins present in foods and fibers are the two main fermentative substrates in the colon. The amount of ingested dietary fiber is a principal factor determining metabolic activity. Recent data have shown that long term dietary pattern influence microbial composition and enterotypes [16]. Finally, he concluded that both probiotics and prebiotics have been explored thoroughly to improve metabolic activity, composition and symbiotic relationships of the gut microbes with the host [17].

### Probiotics as future biotherapeutics to contain diabetes

Dr. M. Balasubramanyam, Assistant Director & Senior Scientist from Madras Diabetes Research Foundation, Chennai delivered his talk on exploring the prospects of novel dietary based strategies including probiotic and prebiotic interventions in the management of Type 2 diabetes by modulating gut hormones. Elaborating on this aspect, he conveyed that with the discovery of gut hormones (the so called incretins viz. GLP-1 and GIP) and their implications in glucose homeostasis, the ‘gut connection’ to type-2 diabetes has now been established beyond peripheral insulin resistance and beta cell failure. In order to substantiate the validity of this hypothesis, he came across with some observations that microbial populations in the gut are different in obese and lean subjects and similarly in diabetic from non-diabetics suggesting that obesity/diabetes may have a different microbial component [18]. Considering the link between alterations in intestinal microbiota and metabolic diseases, Dr. Balasubramanyam claimed that probiotic interventions could serve as potential modulators of gut-flora that change the gut composition in a beneficial manner and exert various health beneficial effects on the host. He further pointed out that certain strains of *Lactobacillus* spp. reported to decrease body fat percentage in healthy volunteers exerted a beneficial effect on the onset of diet-induced obesity by reducing the cell size of white adipose tissues. Realizing the immense potential and scope of using the novel dietary based strategies to effectively control type-2 diabetes, he called upon the researchers that in order to safely and effectively change human gut microflora, the future research endeavors need to be focused to highlight the complex hormonal, immune-modulatory and metabolic mechanisms underlying microbioata-host interactions in different tissues. Furthermore, he stressed that the candidate treatments should be evaluated in well-designed trials with patient-oriented end-points.

In the next presentation, Dr. Anura Kurpad, Professor of Physiology and Nutrition at St. John’s Medical College, Bangalore spoke on the role of gut microflora in meeting the body’s daily requirements of indispensable amino acid (IAA) as per the new WHO/FAO recommendations (2007) which are three fold higher than the previous recommendations. Therefore, the intake of limiting amino acids such as lysine in undernourished populations is likely to be marginally deficient since, these populations also eat a high amount of starch in their predominantly cereal based diets and some starch escapes digestion and absorption in the small intestine and is available for microbial fermentation in the lower reaches of the small intestine and colon for contributing towards the intake of IAAs in the body. Elaborating further on this important subject, Dr. Kurpad enlightened the gathering that the source of nitrogen used for microbial synthesis of IAA could come from urea salvage, or from ingested dietary protein, or endogenous protein secretions. The relationship between requirement and supply of N, and microbial IAA contribution is highly complex, given that urea salvage could be the key to nutritionally significant microbial IAA contribution. He further stated that when N intake is low, the recycling of urea would be of most positive benefit to the body in terms of IAA homeostasis. He further pointed out that although the potential to modulate IAA contribution through the provision of microbial substrate remains unknown, it does have important biological consequences. It might partly explain how undernourished populations living on cereal based low quality protein intakes, evolve alternative mechanisms to meet IAA demands and hence adapt and survive.

Dr. Sharmila Mande, Principal Scientist and Head, TCS Innovation Labs, Tata Consultancy Service Ltd., Pune while presenting the global scenario of malnutrition in her talk highlighted the importance of metagenomics for understanding the role of gut microflora in malnourishment. She further elaborated that by facilitating a direct investigation of the gut microflora, the metagenomics approach aids researchers to obtain a comprehensive understanding of how the variation of the gut microbiome affects human health. She discussed a metagenomic approach for analyzing the difference between gut microbial communities obtained from a malnourished and an apparently healthy child [19]. The analysis of this study showed that malnourished child gut was deficient in several groups like *Lactobacillus* and *Bifidobacteria* but harboured an abundance of enteric pathogens. She further revealed that gut microflora of the malnourished child in addition had a noticeably larger reservoir of antibiotic resistant genes. In long run, identification of such species can help in devising appropriate probiotic strategies for eradicating this menace. Dr. Sesikeran, Director, NIN commented that microbiota of underweight children could be different altogether. Dr. Sharmila further stated that microbiota of Indian children is different from their western counterparts.

### Probiotics in maternal and child health

The interplay between both hereditary and environmental factors play an important role in every stage of development from conception to the early postnatal period with potential long-term effects on mother and child health. The gut microbiota as an environmental factor regulates the development of both metabolic and immune functions during pre and post neonatal life [20].

This session began with the presentation made by Dr. Indrani Ganguli, Head, Institute of obstetrics and Gynaecology, Sir Ganga Ram Hospital, New Delhi who gave a new touch to the probiotic applications by shifting the focus from gut health to vaginal health. She show-cased her interesting findings on the therapeutic role of probiotics in women’s health particularly in the context of management of common vaginal infections. Dr. Ganguli explained in unequivocal terms that absence or depletion of lactobacilli in vagina could be one of the main reasons for bacterial vaginosis and the affetcted cases have a significantly increased risk of HIV, Gonnorrhoea, Chlamydia and Herpes simplex viral infections. She embarked upon that the use of probiotic lactobacilli to prevent infection has a good rationale and an excellent safety record, although, so far only a few strains have been clinically proven to be effective. Hence, she emphasized, it is critically important that strains are characterized and tested clinically using the delivery system of choice (oral tablets, vaginal pessary, dried powder or suspension). She pointed out that besides this, several studies are needed to optimize the defensive properties of the vaginal microbiota. She finally concluded her talk with an optimistic note that the health of many women can be improved by the use of probiotics.

Dr. Ashish Bavdekar, Associate Professor, Department of Pediatrics KEM Hospital, Pune, in his presentation, deliberated at length on the outcome of different clinical meta-analysis studies to demonstrate the efficacy of probiotics against different medical conditions in children. He began his talk by stating that currently probiotics are being administered for various indications like general health improvement, immune enhancement and the prevention and treatment of specific gut related diseases. Dr. Bavdekar further elaborated that the evidence for probiotic efficacy in many such disorders is not so strong, but there are well established benefits in a small number of conditions. Based on the data that emerged from meta-analyses of some clinical studies related to many diarrheal diseases, he contended that probiotics specifically *Lactobacillus rhamnosus* GG, *Lactobacillus reuteri* and *Saccharomyces boulardii* have been shown to significantly shorten the duration of acute diarrhea especially acute rotaviral diarrhea by one day and decreased the number of stools with a beneficial effect starting after 3 days [21]. Similarly, when used as a preventive agent, probiotics have shown reduced incidence of Antibiotic associated Diarrhea (AAD) by an average of 60%. The benefits however, were highly strain and dose dependent. Continuing further debate on the efficacy of probiotics, Dr. Bavdekar commented that probiotics have shown a modest benefit in preventing acute gastrointestinal tract infections in healthy infants and children in child care centers in developed countries. He also referred to a recent community based clinical trial conducted in Kolkata, India wherein *Lactobacillus casei* Shirota was orally fed to 1–5 years old children [22]. The outcome of the study revealed that daily intake of this strain showed a protective efficacy of 14% in preventing acute diarrhea. However, as far as prevention of atopic diseases and IBDs is concerned, there is insufficient scientific evidence to support probiotic efficacy against these conditions. Finally after critical appraisal of the outcome of the meta-analysis studies, he concluded that overall, the scientific data on all these cases have largely remained limited and hence, no recommendations can be made at the moment regarding the use of probiotics in these conditions.

Dr. Pankaj Garg Consultant, Department of Neonatology, Centre for Child Health, Sir Ganga Ram Hospital, New Delhi was the next speaker who delivered a talk on the application of probiotics in pre terms. The major focus of his presentation was on the prospects of probiotic therapy in the management of Necrotizing Enterocolitis (NEC) - a serious GI disorder occurring in neonates. According to Dr. Garg, despite significant advancements in the neonatal care, the mortality resulting from NEC has not improved over the last three decades as can be reflected from high mortality rates which continue ranging from 10–30% [23]. Continuing further, he informed the house that there are only a few interventions which have shown some promise in the treatment of this life threatening disease. In this context, breast milk has been recognized as the best option in the prevention of NEC. However, there is a growing evidence that supplementation of probiotics to preterm infants in NICU may provide sufficient relief to the affected infants [24]. Finding the probiotic therapy an exciting novel strategy, he commented that the rationale for probiotic supplementation of preterm infants is based on the data demonstrating differences in the establishment of intestinal microbiota in preterm infants. In his opinion, administration of probiotics to this vulnerable population may be an effective way to change their gut colonization with the healthy bacteria and advocated exploring the same for disease management in preterms. To further support the efficacy of probiotic supplementation in preterm babies, Dr. Garg referred to the outcome of a recent meta-analysis study carried out in India which provided sufficient scientific evidence to establish the efficacy of probiotics in reducing death and disease in preterm neonates [25]. Based on this study, it was suggested that probiotics should now be offered as a routine therapy for preterm neonates and that additional placebo-controlled trials are not warranted. While concluding his talk, he cautioned that although probiotic supplementation appears to be a promising approach for prevention of NEC in very low birth weight babies, further studies are warranted to address various issues. Currently, there are no well defined regulatory guidelines in the country to ensure the safety and quality of commercially available probiotic preparations. Furthermore, the optimal probiotic combination and dosing strategy also need to be clearly elucidated.

### Future of probiotics in India - roadmap for research

Dr. Sunita Grover, Principal Scientist at NDRI, Karnal, India delivered the first talk in this session and shared her thoughts and vision on reorienting the R&D program on probiotics and shape the future road map in the country in carrying forward this mission holistically through extensive networking and clustering. Since, the diversity of human gut microbiota from different geographical regions, ethnicity, different age groups, populations from different dietary habits and other niches including the traditional fermented foods has not been investigated in India at molecular level and no database is available, it requires extensive efforts from all the cluster partners from different regions in the country to work in tandem towards development of Indian Microbiome database both under healthy and diseased conditions. Furthermore, in India no indigenous probiotic strain is available for commercialization and Indian market is having probiotic products with western strains. Hence, challenge before Indian researchers is to develop indigenous probiotic strains whose specific health claims and safety are scientifically proven and clinically validated in the target population in India. In order to achieve this objective, extensive networking and coordination amongst the researchers working in the area of probiotics, Microbiology, Molecular Biology, Biotechnology, Medical Doctors, Nutrition and Dairy and Food Technology is required to share their expertise to see this dream come true. Dr. Grover also emphasized the need for developing an advanced centre of excellence in probiotic research and product development in the country to make probiotic research highly competitive and at par with the advanced countries. According to her, the centre of excellence should have state of the art infrastructural facilities to carry out work on genomics, transcriptomics, proteomics, human gut models etc. The selection of the strains for functional properties must be based on strategies using ultra modern molecular tools. This centre will be wholly responsible for maintenance of indigenous probiotic strains, build database of gut microbiota originating from various regions of the country, proper documentation of probiotic strains with their functional properties, developing barcoding strategies for tracking probiotic strains besides providing support to researchers and industry working in the area of probiotics. The centre will also look into how to bring indigenous probiotic strains through developing novel functional foods supplemented with these strains to rural population suffering from under nutrition and malnutrition and other gut related diseases. Besides these, India should also have effective regulatory framework to put checks on spurious probiotic products in the country and also to see that the consumers are not misled.

Dr. Rama Chaudhary Professor, Department of Microbiology, AllMS, New Delhi, also delivered a talk in this session and spoke on immune regulation by gut microbiota. She highlighted the importance of gut microbiota in different physiological functions in human gut including immune regulation and mucosal defense. The interaction between this flora and enterocytes is the initiating event in immune-modulation. Furthermore, the gutflora participates in the development of the postnatal immune system as well as oral tolerance and immunity. While elucidating the role of commensal gut microbiota in shaping the gut immunity, Dr. Rama Chadhary elaborated that the gut microbiota acquired during and immediately after birth is necessary for the newborn’s systemic and mucosal immunity. Quoting from some of the previous studies, she suggested that shift in the host microbial community may be associated with number of acute and chronic diseases including inflammatory bowel diseases, obesity, cardiovascular diseases, eczema, vaginal infections etc. She discussed at length how the innate and adaptive immune response in the gut can be modulated effectively to manage these chronic inflammatory diseases by manipulating nutritional based strategies including probiotic interventions. Probiotics can influence the release of specific cytokines by different cell population as well as the crosstalk between the epithelium immune cells in the lamina propria and thus play a fundamental role in mucosal immunity. It has also been observed that the specific probiotic strains can stimulate the secretion of specific cytokines and facilitate the development of naive T-cells towards a particular immune pathway and enhance IgA production and other immunoglobulin in intestinal mucosa. Studies have demonstrated that host protection against gastro-enteric infections is provided by these beneficial bacteria through modulation of pro-inflammatory (e.g. IFNγ and TNFα) and anti-inflammatory (IL-10) cytokines. Thus, these characteristics support the preventive and therapeutic potential of the probiotics or beneficial microbes. She also shared some of her own data on these lines based on the outcome of metagenomic studies to demonstrate immune-modulatory role of probiotics in the management of common gut related inflammatory diseases.

### Novel probiotic foods and formulations-an industrial perspective

This session was chaired by Dr. V. Prakash, Distinguished Scientist of CSIR, Mysore. Dr. Karen Scott, from RINH, University of Aberdeen, Scotland delivered her talk on the impact of diet (carbohydrates) on gut microbiota and consequences for health. In her presentation, she brought to focus dietary substrates playing a key role not only by influencing the gut microbiota but also the health of the host. The colonic microbiota degrades dietary substrates that are undigested or indigestible in the upper GIT, releasing various types of bacterial metabolites, which can either be beneficial or detrimental for gut health. Continuing further, she stated that advances in molecular biology techniques have facilitated detailed analyses of the composition of the bacterial community residing in the lower GIT. Such analyses have indicated that more than 1000 different bacterial species colonize an individual and that although, there is functional consistency in the resident bacterial groups, there is considerable inter-individual variation at the species and strain level. To further put forth her view point, she pointed out that whilst host genome has some impact on the species composition, dietary factors have a greater influence, with specific bacterial groups changing in response to specific dietary interventions. For instance, the number of the Rosenburia /*E. rectale* group increases in the gut following starch supplementation in the diet. Since the metabolic activities of gut bacterial species differ, and can also respond to substrate availability, the consequences for health are dependent on both the specific diet and the composition of the microbiota. The Roseburia / *E. rectale* group are important butyrate producing bacteria, and butyrate is recognized as one of the most important bacterial metabolites for health, being both the preferred energy source for gut epithelial cells and also contributing to apoptosis of the cancerous cells in the gut [26]. Hence, any dietary change that can stimulate the growth of these bacterial species could be beneficial to health. In this context, she also touched upon the prospects of using probiotics and prebiotic supplementation in the gut which are considered beneficial for health and are included in an increasing number of food stuffs. However, before concluding her talk, she cautioned that there can be inter-individual differences in the responses to the same substrate that makes it difficult to generalize the claims relating to health benefits.

### Setting the standards for probiotics: How to meet the requirements and go beyond?

Probiotics provided as natural ingredients to the food, dairy, dietary supplement, infant formula and pharma product have to meet high standards. To maintain high quality, safety, stability and efficacy of probiotic formulations during the entire processing, production and storage chain along with post marketing surveillance is quite challenging and can be achieved only when there is an effective regulatory framework in operation. Realizing the importance and need for having appropriate regulatory standards put in place for probiotics from consumer and industry perspective, Dr. Inge Tarnow, Scientific Advisor, Chr. Hansen A/S Denmark focused her talk on this topic and gave an overview of the global scenario of regulatory standards on probiotics. While discussing the same, she elaborated that probiotics have to be safe, stable and with proven efficacy before administrating them to the consumers. According to her, probiotic efficacy has to be documented in well designed and properly powered randomized controlled human trials that are published in peer reviewed scientific journals. Speaking further on the subject, Dr. Inge advocated that studies conducted according to International Conference of harmonization-Good Clinical Practice (ICH-GCP) are the highest quality standard clinical trials. However, GCP is not mandatory for credible results. She also mentioned that due to stricter regulatory environment particularly in Europe, there is now a demand for high quality clinical data to obtain approval for health claims on food supplements. Furthermore, it is mandatory for probiotic products to be stable throughout shelf life to ensure efficacy. Stability data should be provided right from raw material to the packed consumer product at relevant storage conditions. Safety is of utmost importance and it must be documented and proven. Finally from an industry perspective, probiotic products should preferably be available in different dosage forms as well as customized solutions. This can only be accomplished through innovations and working partnerships. Dr. Inge concluded by suggesting that the industry has to live up to high quality standards and requirement set by regulatory bodies to ensure that consumers receive safe and efficacious products to derive maximal health benefits and specific health claims.

Dr. Nagendra Shah, Professor of Food Science and Technology, University of Hong Kong while delivering his presentation enriched the delegates with an extensive overview on general and specific health benefits and safety of probiotic dairy products. Presenting the overall scenario of the functional food market, he focused on the various types of bacteria being utilized in the world for probiotic products formulation. Dr. Shah specifically mentioned that the most popular probiotic foods such as probiotic yoghurts contain probiotic bacteria as health promoting components and stated that the most common microorganisms used in fermented products belong to the genera lactococci, leuconostocs, pediococci, lactobacillus’and bifidobacteria. He further briefed the house that recently probiotics have been incorporated in drinks and also marketed as supplements including tablets, capsules and freeze dried preparations. Since a large number of probiotic bacteria are ingested orally, such a massive dose can pose a risk to some consumers and hence may require assurance of safety. Besides this, he also discussed the risks associated with some of the probiotic properties used for their selection such as adherence to gut epithelial cells, ability to survive in the gastric conditions and their colonizing potential. He also highlighted the possibility of probiotic translocation from gut to the vital organs through blood causing systemic infections and bacteremia. However, usually translocation is seen in compromised individuals only. Other possible risk factor which figured prominently during the discussions included transfer of antibiotic resistance associated with probiotics to commensal gut flora and the potential gut pathogens which can end up into serious health implications. Hence, safety of probiotic products must be thoroughly checked to rule out all the risk factors by testing both *in-vitro* and *in-vivo* conditions using appropriate models and also clinically.

### Probiotics for adults/elderly

Dr. Colin Hill, Professor of Microbial Food Safety, Microbiology Department and Alimentary Pharmabiotic Centre, University College Cork, Ireland in his presentation shared his recent work on deciphering the possible mechanism of action of probiotics in control of infections. He began his talk by stating that in acute infection, probiotics may augment the protection afforded by commensal gut flora through competitive interactions, direct antagonism of pathogens and or production of antimicrobial factors. He informed that currently, the major goal of his group is to identify the precise mode of action of the specific probiotic effects. In order to analyze the mechanistic basis of the efficacy of selected probiotics against listeriosis, a highly infectious and fatal disease, he and his associates used a mice model system for listeriosis. After mass screening of probiotic strains, they came out with a promising strain i.e. *Lactobacillus salivarius* UCC118 that was very effective in protecting mice from infection. Interestingly, making further inroads into their initiative, he disclosed that the protective effect could be associated with the production of a bacteriocin by the probiotic strain which kills the *Listeria* in the GI tract before it can initiate infection [27] and claimed that identification of a single molecule for probiotic effect was the first, and opened the way for many follow up studies. Further elaborating their work on these lines, Dr. Hill also spoke about extending their work to examine the potential of probiotics and antimicrobial compounds in the treatment of *Clostridium difficile* - one of the main causes of Antibiotic Associated diarrhea. The conventional antibiotic therapy although found effective in mitigating this disease resulted into a decrease in the proportion of sequences assigned to the phyla Firmicutes and Bacteriodes (useful gut flora) with corresponding increase in those assigned to protobacteria [28] leading to collateral damage. He also discussed at length one of the possible means to avoid this collateral damage by application of thiricin CD, a narrow spectrum bacteriocin produced by *Bacillus thuringiensis* DPC6431. It was demonstrated that thuricin CD was equally effective at killing *C. difficile* in the distal colon model, but had no significant impact on the composition of the microbiota. This offers the possibility of developing a targeted approach to eliminating *C. difficile* in the colon without any collateral damage.

Dr. G.P.Talwar, Founder Director, National Institute of Immunology and the present Director Research, Talwar Research Foundation, New Delhi delivered his talk on the role of probiotics in maintaining vaginal and reproductive health. While delivering his presentation, he touched upon the key role played by lactobacilli to maintain acidic conditions in vagina at pH 4 to keep it healthy. Elaborating further, he pointed out that the lactobacilli occurring in vagina prevent the growth of pathogenic bacteria by decreasing the pH through production of D-lactic acid (which unlike L-lactic acid is not metabolized and hence accumulates in the gut and accounts for acidic pH), H_2_O_2_ and bacteriocins with anti-microbial activity. However, Dr. Talwar pointed out that, the resident probiotic lactobacilli may disappear under some conditions such as medication or by any other reason, and the vaginal pH surges beyond 5 and women become susceptible to recurring episodes of reproductive tract infections (RTIs). He also referred to a collaborative study conducted at Sir Ganga Ram Hospital and All India Institute of Medical Sciences wherein out of vaginal swabs collected from 210 women suffering from recurring infections whose vaginal pH was above 5, 126 women were indeed devoid of resident lactobacilli. However, one or more strains of lactobacilli were isolated from vagina of 84 women. To understand this anomaly, quantitative studies were carried out on the D-lactate secreted by strains of lactobacilli isolated from such women and it was found that the amount of D-lactate produced by such strains was too low as compared to those isolated from the vagina of healthy women [29]. Elaborating further on this observation, he explained the outcome of quantitative studies on the amount of D-lactic acid made and secreted by the strains of lactobacilli isolated from infected women. Administration of probiotic lactobacilli of vaginal origin to RTI infected women restored the vaginal pH in the acidic range and protected the subjects from recurrence of RTIs. Dr. Talwar also discussed the efficacy of three of the promising strains of lactobacilli in a clinical study currently in progress at AIIMS and SGRH, New Delhi in women suffering from recurring episodes of RTIs with vaginal pH above pH 5. He informed that that the disease symptoms were cured by intake of a polyherbal microbicide BASANT followed by administration of 3 billion lactobacilli delivered in a veg. capsule.

Dr. Philip Abraham, Consultant Gastroenterologist, P D Hinduja National Hospital, Mumbai focused his talk to the evidence based efficacy of probiotics in gastrointestinal disorders. He however, cautioned the health professionals to be little more vigilant in establishing the efficacy of probiotic strains or formulations in terms of specific health health claims since conflicting conclusions have been recorded from the outcome of clinical studies largely attributed to variations in the agents used, their dose and duration along with evaluation parameter. Dr. Philips reviewed the findings of some investigators in this regard based on a recent meta-analysis which compared the efficacy of probiotics across various diseases [21]. Elaborating further, he particularly referred to some studies wherein probiotics reduced the risk of acute diarrhea of diverse causes by one third, more so among children of antibiotic associated diarrhea by one-half and of travelers’ diarrhea by 8% [30]. However, according to him, their use for prevention of pediatric antibiotic associated diarrhea needs more evidence. He also spoke about one community study conducted in India [22] that showed 14% reduction in acute diarrhea among unprivileged children given *L. casei* strain Shirota. Going further into the efficacy of probiotics health applications from broader perspectives, he stated that meta-analyses and systematic reviews have shown benefit in relief of individual symptoms or in global symptoms in patients with chronic diseases like irritable bowel syndrome [31], although constipation seems to be the symptom with least response to probiotic use. He also pointed out that the VSL#3, a probiotic consortium has been successfully used for the prevention and treatment of chronic pouchitis [32]. He finally concluded his talk by stating that note must be made of the fact that many of the observed effects are strain-specific and conclusions should not be extrapolated and also emphasized that a benefit-versus-potential risk must be considered especially in compromised individuals and in those with serious co-morbidities.

The last speaker in the session Dr. Anders Henriksson, Senior Application Specialist, Dupoint Nutrition & Health, Danisco, Australia focused his talk on probiotics that improve gastrointestinal symptoms. He briefed about the prospects of exploring the probiotic strains of proven functional efficacy in the management of Gastrointestinal (GI) symptoms such as bloating/distension, abdominal pain and constipation which are commonly associated with functional bowel disorders (FBD) such as the irritable bowel syndrome (IBS). According to him, FBD’s are relatively common and it has been estimated that IBS alone affects 3-25% of otherwise healthy people. Milder GI symptoms, not classified as FBD, are even more wide spread. Dr Ander apprised the delegates that Probiotic strains have been used in several clinical studies [33,34] on subjects with GI symptoms and further conveyed that the outcome of such studies suggest that certain probiotic strains alleviate specific symptoms, particularly bloating/distension, abdominal pain and constipation. Elaborating further, he informed that a recent study [35] investigated the effect of *Bifidobacterium lactis* HN019 on the total GI transit time and a range of GI symptoms. Results from this study suggest that HN019 reduces the severity of symptoms by shortening the GI transit time. Results from another study suggest that certain probiotic strains, including *Lactobacillus acidophilus* NCFM, alleviate symptoms of lactose maldigestion. This effect may be mediated by microbial enzymes that aid in digestion of lactose in the upper GI tract. As could be evidenced from these studies along with others, probiotic cultures may be used to alleviate not only milder GI symptoms but also more complex FBD’s. In conclusion, it is anticipated that a dietary supplementation with this type of probiotic cultures would bring benefits to those who are affected by frequent GI symptoms and especially those that are affected by IBS and other FBD’s.

### Panel discussion

This was the last session moderated by Dr. A. K. Srivastava and the panelists included Drs. Seppo Salminen, Colin Hill, C.S. Yajnik, V.K.Batish and Sanjeev Ganguly. The key issues that emerged from the various plenary and technical sessions formed the subject of panel session and discussed at length amongst the panelists and the delegates. After having a thorough brainstorming, some of the crucial issues in terms of priority formed the basis of the following recommendations for further follow up action from PAi for immediate implementation at the governmental level so that it addresses the interest of all the stake holders.

•Since mother and infant relationship creates the first truly probiotic microbes transferred from the mother’s microbiota and breast milk, it is advisable that mothers should be encouraged to consume probiotic products that can play an important role in the development of infants.

•Multicentric well-designed placebo controlled double blind clinical studies on the target local population should be conducted at least at two different locations to establish the functional efficacy of novel probiotic strains or food formulation developed with the same for a specific health claim.

•More extensive efficacy and safety studies need to be carried out on Indian babies before recommending probiotic interventions for preterm neonates due to lack of sufficient clinical data available on these lines.

•The origin of the strains can also influence the efficacy of probiotics against a specific medical condition for a particular host or community. The specific probiotic strain originating from the gut of a particular ethnic community is likely to express its novel physiological function most optimally in the native population due to better colonization and adaptation in the gut relative to a heterologous host community with different food habits. However, there is a need to generate adequate comparative clinical data on efficacy of indigenous strains of probiotic lactobacilli or bifidobacteria in the local target population versus the proven probiotic strains of these organisms originating from western gut to establish differences in relative efficacy of these strains of different gut origin with scientific evidence as proof of this concept.

•Since novel physiological function of probiotics are highly strain specific, it is very crucial that the probiotic organisms must be identified accurately at strain level by using advanced molecular techniques which can discriminate between the genome of closely related species and strains of the same probiotic genera without any ambiguity. In this context, whole genome sequencing of the probiotic strain is now recognized as the most authentic gold standard for probiotic identity.

•The rich microbial diversity of Indian gut needs to be thoroughly investigated in search of novel probiotics due to different food habits, geographical, cultural and anthropological differences and the ethnicity. For achieving this long term objective, it is high time that an all India research coordinated project on these lines should be initiated on priority by identifying different groups from different geographical locations in the country through networking.

• *Lactobacillus*, *Bifidobacterium* and *Saccharomyces boulardii*, which represent only a fraction of the gut microbiota, the key members of probiotic group are the major focus of attention at commercial level from health perspectives. Therefore, other potential organisms such as *Bacteriodes* which constitute the predominant flora in our gut needs to be targeted in search of novel probiotics for broader health applications.

•Selection of potential probiotic strains for product development is an extremely important pre-requisite to establish their efficacy and safety in the target human population. A well designed mass screening strategy based on specific functional biomarkers and *in vitro* tests and *in vivo* animal models needs to be worked out to shortlist the most promising strains for validation in clinical efficacy trials.

•Although, dairy based foods are considered as the best carriers of probiotics to confer their best health promoting functions in the consumers, there is a need to explore other food formulation also for probiotic supplementation to demonstrate their health efficacy. Traditional fermented foods which are already very popular and acceptable amongst the various ethnic groups in the country could be the most attractive targets that can be explored for value addition with proven probiotic strains.

•Since viability and stability of probiotic strains is very crucial to express their health promoting functions during processing of probiotic food formulations and in the gut, new innovative techniques like nano technology and improved food grade encapsulations need to be investigated for better performance in the gut functions.

•Although ICMR-DBT guidelines for evaluation of probiotics in foods have already been put in place in India, there is an urgent need to evolve effective Indian regulatory standards based on these guidelines. In this context, it was felt by all the concerned participating in the conference that PAi should establish immediate contact with FSSAI and expedite the formalities and implementation of regulatory standards on probiotics in the country in harmony with the International standards.

## Conclusion

This conference – a first of its kind initiative in the country from Probiotic Association of India was primarily aimed at fostering probiotic movement in the country by highlighting the enormous potentials of probiotics and probiotic formulations in human health so that the health benefits attributed to them reach the target Indian population. In order to realize this dream, the Conference and the International Symposium on Probiotics brought together a unique mix of several national and International experts on probiotics from leading universities, research institutions, health and nutritional professionals along with food processing and pharma Industry. This scientific conglomeration of almost all the concerned stake holders had made the conference a perfect platform to share knowledge on new Innovations and Emerging Trends in this upcoming area of probiotics. Based on the presentations made by the eminent speakers and young researchers, it is now well established that the proven strains of probiotics with the strongest human health efficacy data duly supported with adequate scientific evidence can promote human health in some of the conditions such as rota virus diarrhoea, antibiotic associated diarrhoea along with *C. difficile* diarrhoea, some other bacterial diarrhoeas and infections besides lactose intolerance. However, numerous questions have still remained unanswered and more information is necessary regarding optimal dosage, duration of therapy origin of the probiotic strain particularly with regard to their colonization and transit in the gut, strain specificity of health claims, regulatory issues related to quality, safety and efficacy of individual and combination of probiotics, continued response rates after withdrawal and the benefits or lack thereof, of adding probiotics to consumer preparations along with probiotic stability and viability at the end of shelf life of the probiotic preparations, including the most important issue of unravelling their mode of action. These constitute some of the most pertinent issues that must be addressed not only for the probiotic supplements as such but also within the context of the specific disease conditions. Nevertheless, irrespective of these unanswered questions, probiotics continue to draw lot of attention amongst the health professionals and consumers find a new ray of hope in probiotics available in all the formats in the market to manage their health care and well being.

## Competing interests

The authors are the office bearers of the Probiotic Association of India (PAi). AKS is the President, VKB the Secretary and SG the Treasurer of PAi. The authors declare that they have no competing interests. HMR is life member of PAi. This comprehensive report is a compilation of the proceedings of the International Symposium and do not reflect the personal views of authors or PAi.

## Authors’ contributions

SG and HMR contributed towards the compilation of the report. VKB conceived the concept and edited the report. AKS gave his critical inputs in terms of improving the overall quality of the manuscript. All authors read and approved the final manuscript.

## References

[B1] Prajapati JB Khedkar CD Chitra J Suja S Mishra V Sreeja V Patel RK Ahir VB Bhatt VD Sajnani MR Sajnani MR Jakhesara SJ Koringa PG Joshi CG Whole-genome shotgun sequencing of an Indian-origin Lactobacillus helveticus strain, MTCC 5463, with probiotic potential J Bacteriol 2011 193 16 4282 4283 21705605 10.1128/JB.05449-11 PMC3147707

[B2] Duary RK Batish VK Grover S Expression of the atpD gene in probiotic *Lactobacillus plantarum* strains under in vitro acidic conditions using RT-qPCR Res Microbiol 2010 161 5 399 405 20416373 10.1016/j.resmic.2010.03.012

[B3] Duary RK Batish V Grover S Relative gene expression of bile salt hydrolase and surface proteins in two putative indigenous *Lactobacillus plantarum* strains under in vitro gut conditions Mol Biol Rep 2012 39 3 2541 2552 21674190 10.1007/s11033-011-1006-9

[B4] Duary RK Bhausaheb MA Batish VK Grover S Anti-inflammatory and immunomodulatory efficacy of indigenous probiotic *Lactobacillus plantarum* Lp91 in colitis mouse model Mol Biol Rep 2012 39 4 4765 4775 21947851 10.1007/s11033-011-1269-1

[B5] Duary RK Rajput YS Batish VK Grover S Assessing the adhesion of putative indigenous probiotic lactobacilli to human colonic epithelial cells Indian J Med Res 2011 134 664 671 22199106 10.4103/0971-5916.90992 PMC3249965

[B6] Kumar H Rangrez A Dayananda K Atre A Patole M Shouche Y *Lactobacillus plantarum* (VR1) isolated from an Ayurvedic medicine (Kutajarista) ameliorates in vitro cellular damage caused by Aeromonas veronii BMC Microbiol 2011 11 1 152 21707995 10.1186/1471-2180-11-152 PMC3145568

[B7] Hoffman FA Heimbach JT Sanders ME Hibberd PL Executive summary: scientific and regulatory challenges of development of probiotics as foods and drugs Clin Infect Dis 2008 46 2 S53 S57 18181723 10.1086/523342

[B8] Culligan E Hill C Sleator R Probiotics and gastrointestinal disease: successes, problems and future prospects Gut Pathog 2009 1 1 19 19930635 10.1186/1757-4749-1-19 PMC2789095

[B9] Rautava S Luoto R Salminen S Isolauri E Microbial contact during pregnancy, intestinal colonization and human disease Nat Rev Gastroenterol Hepatol 2012 9 10 565 576 22890113 10.1038/nrgastro.2012.144

[B10] Cabrera-Rubio R Collado MC Laitinen K Salminen S Isolauri E Mira A The human milk microbiome changes over lactation and is shaped by maternal weight and mode of delivery Am J Clin Nutr 2012 96 3 544 551 22836031 10.3945/ajcn.112.037382

[B11] van Loveren H Sanz Y Salminen S Health claims in Europe: probiotics and prebiotics as case examples Annu Rev Food Sci Technol 2012 3 247 261 22224554 10.1146/annurev-food-022811-101206

[B12] Lozupone CA Stombaugh JI Gordon JI Jansson JK Knight R Diversity, stability and resilience of the human gut microbiota Nat 2012 489 7415 220 230 10.1038/nature11550 PMC3577372 22972295

[B13] Vyas U Ranganathan N Probiotics, prebiotics and synbiotics: Gut and beyond Gastroenterol Res Pract 2012 2012 1 16 10.1155/2012/872716 PMC3459241 23049548

[B14] Atarashi K Tanoue T Shima T Imaoka A Kuwahara T Momose Y Cheng G Yamasaki S Saito T Ohba Y Taniguchi T Takeda K Hori S Ivanov II Umesaki Y Itoh K Honda K Induction of colonic regulatory T cells by indigenous Clostridium Species Sci 2011 331 6015 337 341 10.1126/science.1198469 PMC3969237 21205640

[B15] Guarner F Bourdet-Sicard R Brandtzaeg P Gill HS McGuirk P van Eden W Versalovic J Weinstock JV Rook GA Mechanisms of disease: the hygiene hypothesis revisited Nat Clin Pract Gastroenterol Hepatol 2006 3 275 284 16673007 10.1038/ncpgasthep0471

[B16] Wu GD Chen J Hoffmann C Bittinger K Chen YY Keilbaugh SA Bewtra M Knights D Walters WA Knight R Sinha R Gilroy E Gupta K Baldassano R Nessel L Li H Bushman FD Lewis JD Linking long-term dietary patterns with gut microbial enterotypes Sci 2011 334 6052 105 108 10.1126/science.1208344 PMC3368382 21885731

[B17] McNulty NP Yatsunenko T Hsiao A Faith JJ Muegge BD Goodman AL Henrissat B Oozeer R Cools-Portier S Gobert G Chervaux C Knights D Lozupone CA Knight R Duncan AE Bain JR Muehlbauer MJ Newgard C Heath AC Gordon JI The impact of a consortium of fermented milk strains on the gut microbiome of gnotobiotic mice and monozygotic twins Sci Transl Med 2011 3 106 106 10.1126/scitranslmed.3002701 PMC3303609 22030749

[B18] Larsen N Vogensen FK van den Berg FWJ Nielsen DS Andreasen AS Pedersen BK Al-Soud WA Sørensen SJ Hansen LH Jakobsen M Gut microbiota in human adults with type 2 diabetes differs from non-diabetic adults PLoS One 2010 5 2 e9085 20140211 10.1371/journal.pone.0009085 PMC2816710

[B19] Gupta S Mohammed M Ghosh T Kanungo S Nair G Mande S Metagenome of the gut of a malnourished child Gut Pathog 2011 3 1 7 21599906 10.1186/1757-4749-3-7 PMC3115890

[B20] Sanz Y Gut microbiota and probiotics in maternal and infant health Am J Clin Nutr 2011 94 6 2000S 2005S 21543533 10.3945/ajcn.110.001172

[B21] Ritchie ML Romanuk TN A meta-analysis of probiotic efficacy for gastrointestinal diseases PLoS One 2012 7 4 e34938 22529959 10.1371/journal.pone.0034938 PMC3329544

[B22] Sur D Manna B Niyogi SK Ramamurthy T Palit A Nomoto K Takahashi T Shima T Tsuji H Kurakawa T Takeda Y Nair GB Bhattacharya SK Role of probiotic in preventing acute diarrhoea in children: a community-based, randomized, double-blind placebo-controlled field trial in an urban slum Epidemiol Infect 2011 139 06 919 926 20670468 10.1017/S0950268810001780

[B23] Grosfeld JL Chaedt M Molinari F Increased risk of necrotizing enterocolitis in premature infants with patent ductus arteriosus treated with indomethacin Ann Surg 1996 224 3 350 357 8813263 10.1097/00000658-199609000-00011 PMC1235380

[B24] Lin HC Su BH Chen AC Lin TW Tsai CH YehTF OW Oral probiotics reduce the incidence and severity of necrotizing enterocolitis in very low birth weight infants Paediatr 2005 115 1 1 4 10.1542/peds.2004-1463 15629973

[B25] Deshpande G Rao S Patole S Bulsara M Updated meta-analysis of probiotics for preventing necrotizing enterocolitis in preterm neonates Paediatr 2010 125 5 921 930 10.1542/peds.2009-1301 20403939

[B26] Walker AW Ince J Duncan SH Webster LM Holtrop G Ze X Dominant and diet-responsive groups of bacteria within the human colonic microbiota ISME J 2011 5 220 230 20686513 10.1038/ismej.2010.118 PMC3105703

[B27] Corr SC Li Y Riedel CU O’Toole PW Hill C Gahan CGM Bacteriocin production as a mechanism for the antiinfective activity of Lactobacillus salivarius UCC118 Proc Natl Acad Sci 2007 104 18 7617 7621 17456596 10.1073/pnas.0700440104 PMC1863472

[B28] Rea MC Sit CS Clayton E O’Connor PM Whittal RM Zheng J Vederas JC Ross RP Hill C Thuricin CD, a posttranslationally modified bacteriocin with a narrow spectrum of activity against Clostridium difficile Proc Natl Acad Sci 2010 107 20 9352 9357 20435915 10.1073/pnas.0913554107 PMC2889069

[B29] Garg KB Ganguli I Kriplani A Lohiya NK Thulkar J Talwar GP Metabolic properties of lactobacilli in women experiencing recurring episodes of bacterial vaginosis with vaginal pH ≥ 5 Eur J Clin Microbiol Infect Dis 2010 29 1 123 125 19802749 10.1007/s10096-009-0818-1

[B30] Sazawal S Hiremath G Dhingra U Malik P Deb S Black RE Efficacy of probiotics in prevention of acute diarrhoea: a meta-analysis of masked, randomised, placebo-controlled trials Lancet Infect Dis 2006 6 6 374 382 16728323 10.1016/S1473-3099(06)70495-9

[B31] Moayyedi P Ford AC Talley NJ Cremonini F Foxx-Orenstein AE Brandt LJ Quigley EMM The efficacy of probiotics in the treatment of irritable bowel syndrome: a systematic review Gut 2010 59 3 325 332 19091823 10.1136/gut.2008.167270

[B32] Holubar SD Cima RR Sandborn WJ Pardi DS Treatment and prevention of pouchitis after ileal pouch-anal anastomosis for chronic ulcerative colitis Cochrane Database Syst Rev 2010 6 CD001176 20556748 10.1002/14651858.CD001176.pub2

[B33] Kaptchuk TJ Friedlander E Kelley JM Sanchez MN Kokkotou E Singer JP Kowalczykowski M Miller FG Kirsch I Lembo AJ Placebos without deception: A randomized controlled trial in irritable bowel syndrome PLoS One 2010 5 12 e15591 21203519 10.1371/journal.pone.0015591 PMC3008733

[B34] Ford AC Moayyedi P Meta-analysis: factors affecting placebo response rate in the irritable bowel syndrome Aliment Pharmacol Ther 2010 32 2 144 158 20412064 10.1111/j.1365-2036.2010.04328.x

[B35] Waller PA Gopal PK Leyer GJ Ouwehand AC Reifer C Stewart ME Miller LE Dose–response effect of *Bifidobacterium lactis* HN019 on whole gut transit time and functional gastrointestinal symptoms in adults Scand J Gastroenterol 2011 46 9 1057 1064 21663486 10.3109/00365521.2011.584895 PMC3171707

